# Fennel Seed Biochar: A Sustainable Approach for Methylene Blue Removal from Aqueous Solutions

**DOI:** 10.3390/ma17174350

**Published:** 2024-09-02

**Authors:** Dorota Paluch, Aleksandra Bazan-Wozniak, Agnieszka Nosal-Wiercińska, Judyta Cielecka-Piontek, Robert Pietrzak

**Affiliations:** 1 Department of Applied Chemistry, Faculty of Chemistry, Adam Mickiewicz University, Uniwersytetu Poznańskiego 8, 61-614 Poznan, Poland; dorota.paluch@amu.edu.pl (D.P.); aleksandra.bazan@amu.edu.pl (A.B.-W.); 2 Department of Analytical Chemistry, Institute of Chemical Sciences, Faculty of Chemistry, Maria Curie-Skłodowska University, Maria Curie-Skłodowska Sq, 3, 20-031 Lublin, Poland; agnieszka.nosal-wiercinska@mail.umcs.pl; 3 Department of Pharmacognosy, Faculty of Pharmacy, Poznan University of Medical Sciences, Rokietnicka 3, 60-806 Poznan, Poland; jpiontek@ump.edu.pl

**Keywords:** biochar, direct activation, methylene blue, adsorption study, XPS analysis

## Abstract

In this study, biochars were produced from by-products of the herbal industry, specifically fennel seeds (*Foeniculum vulgare*), through direct activation by carbon dioxide at two different temperatures. The biochar samples were comprehensively analysed. Additionally, adsorption studies were conducted for methylene blue. The resulting adsorbents exhibited a specific surface area ranging from 2.29 to 14.60 m^2^/g. The resulting materials displayed a basic character on their surface. The constants for adsorption models were determined for each dye as well as thermodynamic parameters and the kinetics of the process. The sorption capacities of methylene blue for the samples exhibited a range of 22 to 43 mg/g. The adsorption kinetics of the dye on the biochar materials were found to follow a pseudo-second-order model, with the adsorption process best described by the Langmuir isotherm for the DA-800 sample and the Freundlich isotherm for the DA-750 sample. This indicates the development of a monolayer adsorbate on the biochar surfaces. The efficacy of the adsorption process in aqueous solutions of methylene blue was found to increase with rising temperature. Furthermore, based on thermodynamic studies, the adsorption process was found to be spontaneous and endothermic.

## 1. Introduction

**Figure 1:**
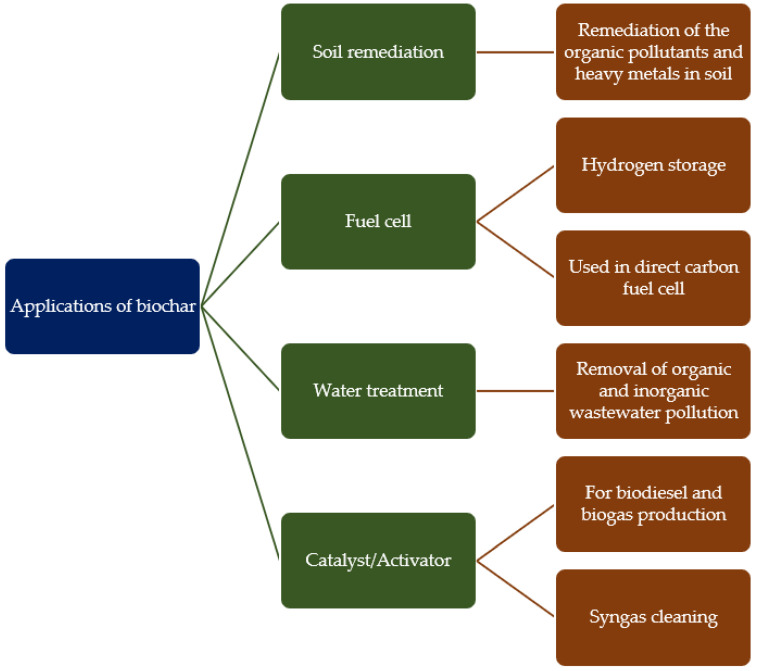
Most popular applications of biochar.

Biochar is a porous, carbon-rich solid material produced by the thermal decomposition of biomass in anaerobic conditions. It is characterised by a high degree of aromatisation and resistance to decomposition [1]. Interest in biochar has grown significantly in recent years due to its versatility in agricultural and industrial applications. [2]. The physicochemical properties of biochar, including its elemental composition, porosity, and pH, are dependent upon the source of the biomass from which it is derived, and the production process employed. These factors influence the suitability of the material for a range of applications [3]. Over the centuries, biochar has found many different uses (Figure 1). In the context of agriculture, the application of biochar has been demonstrated to enhance soil quality by reducing the degradation of nutrients [4]. Furthermore, biochar is employed for the treatment of waste, effectively removing a range of pollutants, including organic and inorganic substances, as well as dyes from textiles [5]. Moreover, it can be employed as a renewable energy source through its high carbon content and as a catalyst for biodiesel and biogas production [6].

Biomass can be converted into renewable fuels through the application of biochemical and thermochemical techniques. In general, the production of biochar at high temperatures (300–900 °C) results in a highly aromatic nature, with a well-structured carbon layer [7].

Currently, biochar serves as a dependable alternative sorbent to activated carbon, carbon nanotubes, and graphene, offering comparable adsorption capacities for both organic and inorganic pollutants. [1]. Consequently, the primary advantages of biochar synthesis are the mitigation of waste-related issues, the expedient and cost-effective preparation of the material, and the remediation of wastewater [8,9]. The utilisation of waste biomass for the generation of biochar represents a further exemplification of the concept of resource reuse.

Herbal plants are characterised by their diverse content of biologically active substances, which makes them widely used in industries. The content of biologically active substances in herbal plants includes various types of chemical compounds such as anthocyanins, flavonoids, glycosides, alkaloids, saponins, and tannins. Herbal raw materials include seeds (*semen*), rhizomes (*rhizoma*), leaves (*folium*), roots (*radix*), flowers (*flos*), fruits (*fructus*), and bark (*cortex*) [10]. The utilisation of agricultural waste as precursors for activated carbon has also been identified as a renewable and relatively cost-effective approach, with the potential to effectively transform waste into a valuable resource. Any inexpensive material with a high carbon and low inorganic content can be employed as a precursor for activated carbon production [11,12]. While all plants are regarded as biomass, various forms of biomass are frequently employed as a source for the production of or conversion into useful products. For example, agricultural biomass fibre, such as kenaf core fibre, was utilised to create carbon adsorbent [13]. Additionally, fruit waste, including citrus peel waste and exhausted grape marc from the food processing industry, is employed as biomass for the production of either biofuels or biochar [14].

Fennel is native to the Mediterranean and West Asia. It is now cultivated worldwide, especially in Mediterranean countries. The annual production of fennel is around 600,000 tonnes and is associated with the herbaceous waste, including batches of seeds that have not passed quality control [15]. Fennel seeds can serve as a precursor for producing carbon adsorbents, specifically biochar, because of their properties, distinctive structure, and widespread availability.

The effects of unsustainable material development and poor resource management are driving the adoption of concepts such as circular economy in waste management. In industry, the pursuit of cost-effective and accessible materials, coupled with comprehensive life cycle assessments, has consistently driven efforts towards sustainability and the advancement of greener chemistry. This research presents a comprehensive analysis of the production and physicochemical characteristics of two biochars, obtained through one-step direct activation of a less-explored source: fennel seeds. The material employed is waste from the herbal industry, which represents an environmentally friendly approach to the production of carbon bioadsorbents. The novelty of the research lies in the optimisation of the production of biochar using fennel seeds as a precursor, aiming to create affordable biochar adsorbents with effective sorption capabilities for waterborne pollutants, particularly organic dyes.

## 2. Materials and Methods

### 2.1. Precursor and Biochar Preparation

The synthesis of biochar was achieved through the utilisation of fennel seeds as the precursor. These seeds, which were below quality control standards, were regarded to be residues from herb production. Subsequently, the precursor was subjected to direct activation in a conventional furnace in a carbon(IV) oxide atmosphere with a flow rate of 190 cm^3^/min for 60 min at one of two temperature options: 1023.15 K (750 °C) or 1073.15 K (800 °C). The materials obtained were subsequently designated with the symbols DA-750 and DA-800. The resulting materials were then dried until a solid mass was achieved, after which they were sieved through a 0.09 mm sieve.

### 2.2. Characterisation of Biochar

The textural properties of the obtained samples were examined through nitrogen adsorption/desorption isotherm analysis at 77.15 K, utilising an AutosorbiQ analyser from Quantachrome Instruments. Before the analysis, the biochar samples were subjected to vacuum degassing at 573.15 K for 12 h. The Brunauer, Emmett, Teller (BET) method was employed to determine the surface area of the adsorbents based on the nitrogen adsorption isotherm. The total pore volume (V_T_) was calculated by measuring the nitrogen volume adsorbed at a relative pressure of p/p_0_ = 0.99, which is obtained by dividing the equilibrium pressure by the saturation pressure. This value corresponds to the volume of adsorbed liquid nitrogen at a specific temperature. The value of the average pore diameter (D) was computed using the following formula: D = 4 V_T_/S_BET_, where S_BET_ represents the surface area of the biochar samples. It was assumed that the pores were cylindrical.

The Boehm method was employed to determine the surface oxygen functional groups with both basic and acidic properties of obtained biochar samples. To measure the pH of the aqueous extracts from the adsorbent samples, 0.1 g of biochar was precisely weighed and mixed with 10 cm^3^ of distilled water. The resulting suspension was continuously stirred for 24 h to reach equilibrium.

The iodine number is measured following the guidelines of the ASTM D4607-94 method [16].

A Phoibos 150 NAP analyser (Specs) was used to perform X-ray photoelectron spectroscopy analysis. The vacuum level during the analysis was approximately 5 × 10^−9^ mbar. The samples were irradiated with non-monochromatic Mg Kα radiation.

### 2.3. Adsorption Studies

Methylene blue, an organic dye, was chosen as the model pollutant for adsorption studies. Each 25 mg biochar sample was added to a 50 cm^3^ aqueous dye solution with an initial concentration ranging from 5 to 30 mg/dm^3^. The mixtures were stirred at room temperature (295.15 ± 1 K) on a laboratory shaker at 250 rpm for 24 h. Afterwards, around 5 cm^3^ of solution from each mixture was extracted and centrifuged using a laboratory centrifuge. The absorbance of the solutions was then determined spectrophotometrically at a wavelength of 665 nm with a UV-VIS spectrophotometer. This approach was used for all adsorption experiments. The adsorption capacities (1) of the biochar were calculated using the given equation:

(1) $$q_{e}=\frac{C_{0-}C_{e}}{m}\times V$$

C_0_—the initial concentrations (mg/dm^3^) of the dye in solution; C_e_—the equilibrium concentrations (mg/dm^3^) of the dye in solution; m—the mass of the biochar (g); V—the volume of the solution (dm^3^).

To determine a suitable model for the adsorption of dye on a biochar, the linear forms of the Langmuir and Freundlich equations were used. The Langmuir equation is based on the assumption that the surface of the adsorbent has a finite number of energetically equal active sites capable of adsorbing a single adsorbate molecule [17]. The following linear equation describes the Langmuir isotherm (2):

(2) $$\frac{1}{q_{e}}=\frac{1}{q_{max}}+\frac{1}{K_{L}q_{m}}\times \frac{1}{C_{e}}$$

q_e_—the equilibrium amount of adsorbed dye (mg/g); K_L_—Langmuir equilibrium constant (dm^3^/mg); q_max_—the maximum adsorption capacity of the adsorbent (mg/g).

The Freundlich model states that there can be no more adsorbed species than active sites, and that the layer formed on the adsorbing surface can form more [18]. The description of this model is the following Equation (3):

(3) $${logq}_{e}={logK}_{F}+\frac{1}{n}{logC}_{e}$$

K_F_—Freundlich equilibrium constant (mg/g(dm^3^/mg)^1/n^); 1/n—the adsorption intensity constant.

For further research, the initial concentration of methylene blue was 20 mg/dm^3^.

The effect of the pH value (pH 3–11) of the aqueous solution of methylene blue on the sorption capacity of the prepared adsorbents was determined. Measurements were carried out on 25 mg samples of biochar. At a predetermined pH, each sample was immersed in 50 cm^3^ of aqueous dye solution. The pH of the dye solution was adjusted by adding 0.1 M HCl or 0.1 M NaOH. The mixtures were stirred under the same conditions as described above.

Moreover, the study examined the influence of process temperature on the adsorption of aqueous solutions of methylene blue. The initial experiment was conducted using test samples with a weight of 25 mg for each individual sample. These samples were immersed in a fixed aqueous solution with a volume of 50 cm^3^. The samples were agitated for 24 h at 298.15, 318.15, and 338.15 K using a laboratory shaker at 250 rpm for a duration of 24 h. Following this period, approximately 5 cm^3^ of solution was extracted from each solution, subjected to centrifugation in a laboratory centrifuge, and then its absorbance was examined spectrophotometrically.

The thermodynamic parameters were calculated using the following formulas:

$$∆G^{0}=-RTlnK_{d}\;∆G^{0}=∆H^{0}-T∆S^{0}\;lnK_{d}=\frac{∆S^{0}}{R}+\frac{∆H^{0}}{RT}$$

ΔG^0^—Gibbs free energy, R—universal constant (8.314 J/mol × K), T—temperature (K), ΔH^0^—enthalpy change, ΔS^0^—entropy change, K_d_—thermodynamic equilibrium constant.

In order to ascertain the kinetics of dye adsorption on the biochar samples, a quantity of 25 mg of the sample was immersed with 50 cm^3^ of dye solution at a fixed concentration. The bottle was positioned in a laboratory shaker at room temperature and at a speed of 300 rpm/min. Subsequently, spectrophotometric measurements were taken over a period of 420 min. Two models were used for the purpose of data analysis: the pseudo-first-order model (5), the pseudo-second-order (6) [19], and intraparticle diffusion (7) [20]:

$$\log\left(q_{e}-q_{t}\right)=logq_{e}-\frac{k_{1}}{2.303}t\;\frac{t}{q_{t}}=\frac{1}{k_{2}{q_{e}}^{2}}+\frac{t}{q_{e}}\;q_{t}=k_{id}t^{1/2}+C$$

q_e_—the equilibrium amount of adsorbed dye (mg/g); q_t_—the amount of adsorbed dye over time (mg/g); t—the process time (min); k_1_—the pseudo-first-order adsorption constant (1/min); k_2_—the pseudo-second-order adsorption constant (g/mg × min); k_id_—the intraparticle diffusion constant (mg/g × min^1/2^); C—the boundary layer constant (mg/g).

## 3. Results and Discussion

### 3.1. Physiochemical Characterisation of the Biochar

#### Textural Parameters

**Figure 2:**
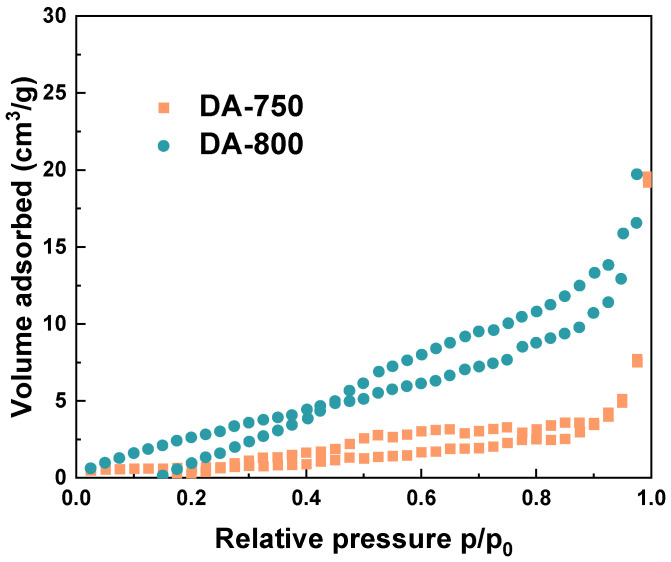
Low-temperature N_2_ adsorption/desorption isotherms of obtained biochar.

The nitrogen adsorption/desorption analysis produces distinct adsorption and desorption curves that correspond to different pore types and structural characteristics. The curves form a hysteresis loop, as they do not align perfectly. The shape and timing of this loop are essential for identifying pore shapes and are, therefore, crucial for the analysis. Figure 2 illustrates the low-temperature nitrogen adsorption/desorption isotherms of the obtained biochar samples. According to the IUPAC classification, these can be categorised as type II (DA-800) and type III (DA-750) with a small hysteresis loop type H3 [21,22]. The Type H3 hysteresis loop is attributed to the formation of wedge-shaped pores resulting from the loose stacking of flaky particles [23]. The Type II isotherm is concave, then almost linear and finally convex, and is typical for macroporous sorbents. However, the Type III isotherm is convex over the entire range. It also indicates weak sorbent–sorbate interactions [24].

Table 1 presents the textural parameters of the obtained biocarbon materials. The results indicate that the materials exhibit a markedly inferior development of the specific surface area. These sorbents exhibit a meso–macroporous structure, which is confirmed by both the BET method and textural parameters. The average pore diameter of sample DA-750 is approximately twice that of sample DA-800, with a value of 53.01 nm compared to 25.75 nm, respectively. The sample DA-800, obtained by direct activation of fennel seeds with carbon dioxide at 1073.15 K, has a specific surface area that is almost seven times greater than that of a sorbent obtained at a temperature that is lower by 50 K. Furthermore, this material has a pore volume that is three times greater than that of the DA-750 sample. The iodine number value of the DA-800 sample is 12 mg/g higher than that of the sample similarly obtained at a lower temperature, indicating superior sorption properties of this material. Furthermore, it can be observed that an increase in the activation temperature resulted in a slight increase in the ash content of the material, amounting to a mere 2% increase.

A study conducted by Rajapaksha et al. demonstrated that the steam activation of burcucumber at 973.15 K for a period of 45 min resulted in the production of a material with a specific surface area of 7 m^2^/g [25]. Furthermore, biochar derived from buffalo-weed through direct activation under a restricted supply of air at 973.15 K exhibited a specific surface area of 9.25 m^2^/g [26]. The values obtained for these samples are higher than those obtained for sample DA-750. However, heating the starting material to a higher temperature (1073.15 K) resulted in a specific surface area more than twice that of the biochar obtained from burcucumber and buffalo-weed. It is notable that the biochar obtained under analogous conditions to those employed for the DA-800 sample, using caraway seeds as the precursor, exhibited a specific surface area of 10 m^2^/g [27]. This value is approximately one-third less than that obtained for fennel seed biochar.

In conclusion, both obtained materials exhibit a poorly developed porous structure. Nevertheless, the surface of sample DA-800 exhibits more favourable parameters following the adsorption process than sample DA-750. Increasing the activation temperature by only 50 K increased the specific surface area development by up to seven times. In addition, the results obtained do not differ from the values determined for the reported adsorbents obtained from other types of waste materials.

**Figure 3:**
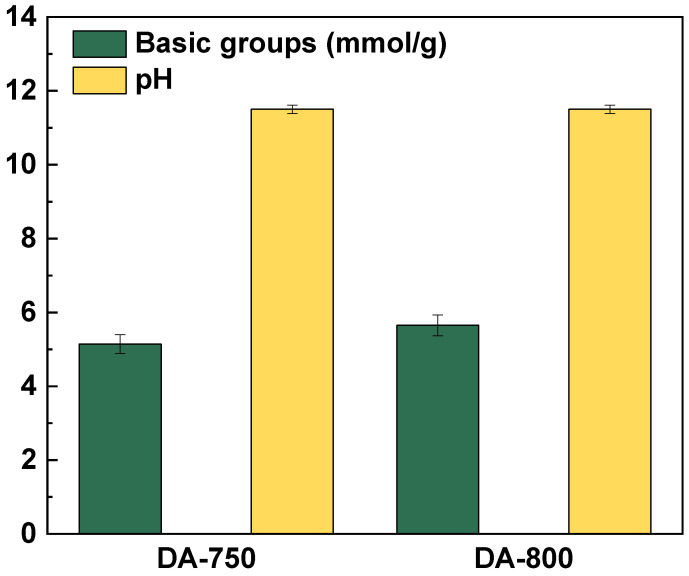
Content of oxygen functional groups on the surface of obtained biochar.

Figure 3 presents the results of the Boehm method. The test indicates that both sorbents obtained show similar surface properties, with no acidic oxygen groups and a content of basic groups of 5.14 and 5.65 mmol/g, respectively, for samples DA-750 and DA-800. The results are further supported by the alkaline pH of the aqueous extract of these sorbents, which is 11.5 in both cases. The absence of acid groups on the surface of the materials obtained can be ascribed to the activation method employed, specifically direct activation with carbon(IV) oxide, which has been demonstrated to favour the formation of basic groups. The same outcomes were observed in other studies [27,28].

**Figure 4:**
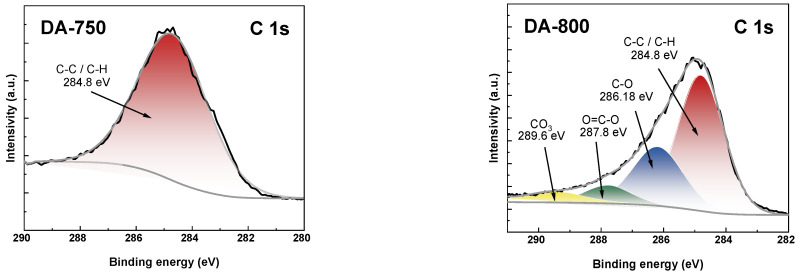
The XPS oxygen (C 1s) spectra of obtained biochar.

**Figure 5:**
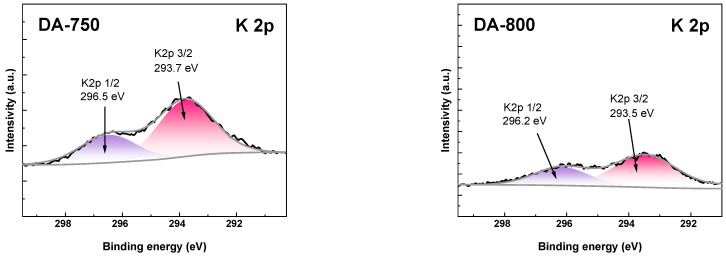
The XPS potassium (K 2p) spectra of obtained biochar samples.

**Figure 6:**
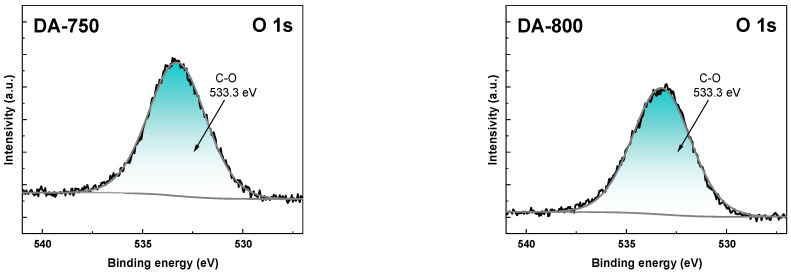
The XPS oxygen (O 1s) spectra of obtained biochar.

To study detailed surface composition, the high-resolution XPS spectra of C 1s, K 2p and O 1s was shown in Figure 4, Figure 5 and Figure 6. As shown in Figure 4, the C 1s spectra of DA-800 sample could be resolved into three to four peaks at 284.8, 286,18, 287.8, and 289.6 eV assigned to aromatic C-C/C-H, aromatic C-O, carboxylic O-C=O, and CO_3_ bond, respectively [29].

The presence of carbonates on the surface of the obtained biochar sample is due to the use of carbon(IV) oxide as an activator. In contrast, in the case of sample DA-750, only a signal at 284.8 eV is visible in the C 1s spectra, originating from the C-C/C-H bond. This low speciation of carbon atoms in the DA-750 sample is due to its poorly developed specific surface area. XPS analysis allowed the detection of potassium in the obtained samples. In Figure 5, peaks originating from K 2p 1/2 and K 2p 3/2 can be distinguished. The presence of potassium on the surface of the adsorbents tested can be attributed to the organic starting material. The O 1s spectra (Figure 6) could be resolved into one peak at 533.3 eV and is attributed to hydroxyl groups (C-OH) [30].

Table 2 presents the elemental contents, expressed as percentage atomic concentration (% At), for the biochar obtained through X-ray photoelectron spectroscopy (XPS) analysis. A comparison of the data presented indicates that the adsorbent obtained by direct activation with carbon(IV) oxide at the higher temperature exhibits a higher oxygen content and a lower carbon and potassium content than the sample obtained at a temperature 50 K lower. The application of a higher activation temperature resulted in enhanced development of the specific surface area of the DA-800 adsorbent which, in turn, led to an increase in the oxygen content on its surface. This, in turn, contributed to a reduction in the concentration of other elements on its surface. The relatively low oxygen content observed on the surface of the materials under investigation can be attributed to the activation method employed. It has been demonstrated that the activation of sorbents with carbon(IV) oxide results in the formation of materials with low polarity and a consequently low O/C ratio [1].

### 3.2. Adsorption Study

**Figure 7:**
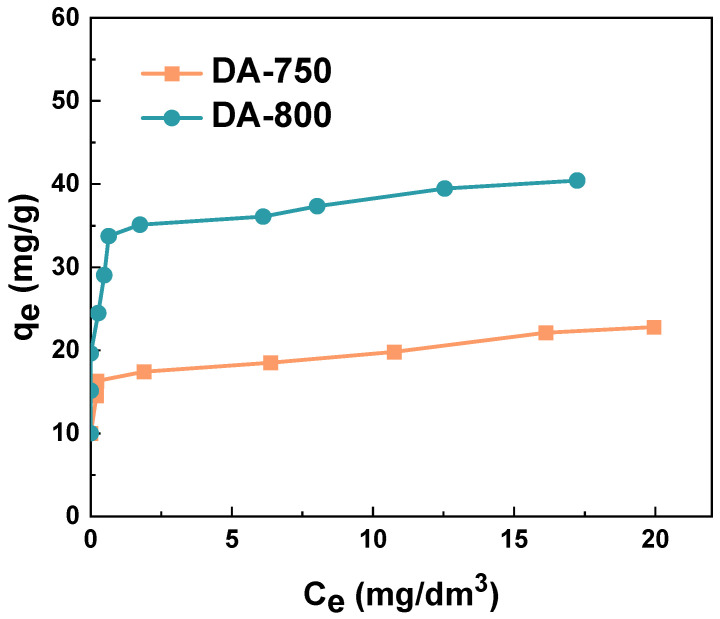
Isotherm of methylene blue adsorption on obtained biochar (volume of dye solution: 50 dm^3^; dye concentration: 5–30 mg/dm^3^, shaking speed: 250 rpm/min, time of adsorption: 24 h, temperature: 295.15 ± 1 K).

Figure 7 illustrates the correlation between the concentration of methylene blue in an aqueous solution and the sorption capacity of the prepared biochar. As illustrated in Figure 7, an increase in the concentration of the dye results in a corresponding enhancement in the sorption capacity of the studied samples. This relationship was observed for both adsorbents, indicating that with increasing dye concentration, the efficacy of the interactions/collisions between the adsorbate and adsorbent increases, resulting in enhanced sorption capacity [31]. The sorption capacities of the samples ranged from 22 to 43 mg/g. The enhanced sorption capacity of sample DA-800 is attributed to its relatively more developed surface area.

The sorption capacities obtained for methylene blue can be compared with the values obtained for biochars prepared from other precursors. The biochar obtained from wheat straw had a sorption capacity for the test dye of 12 mg/g [32]. This figure is approximately twice as low as that obtained for biochar DA-750. The reed-derived biochar demonstrated a capacity towards methylene blue of 19 mg/g. However, following the activation of the material with 2M HNO₃, this capacity increased to 33 mg/g, which remains lower than that of the DA-800 biochar subjected to one-step activation with carbon dioxide [33]. The adsorbent obtained from pig manure at a low temperature of 773.15 K has a sorption capacity towards methylene blue of 53 mg/g, a value exceeding that obtained for sample DA-800 by 10 mg/g [34]. Moreover, subjecting another waste material, rabbit manure, to the same thermal treatment conditions results in the production of a sorbent with a sorption capacity of up to 104 mg/g [34]. This evidence supports the assertion that the selection of the precursor is a crucial factor in the biochar production process.

**Figure 8:**
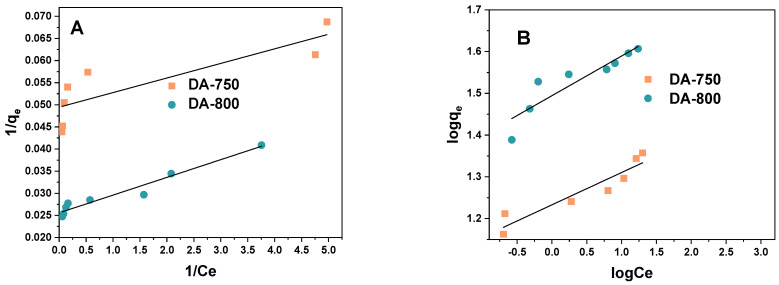
Linear fitting for methylene blue on obtained biochar to (**A**) Langmuir model and (**B**) Freundlich model.

The relationship between the data obtained from the adsorption process and the theoretical or empirical equations can facilitate the acquisition of further insights into the adsorption of dye in relation to the sorbent, thus, providing a deeper understanding of the underlying processes. The linear fit of the experimental data to the Langmuir and Freundlich models is shown in Figure 8. The applicability of two isotherm equations was evaluated by calculating the parameters (Table 3).

A detailed examination of the correlation coefficients (R^2^ and Adj^2^) presented in Table 3 shows that, in the case of sample DA-750, the Freundlich model (R^2^ = 0.886) provides a better fit than the Langmuir model (R^2^ = 0.724). However, in the case of sample DA-800, the Langmuir isotherm demonstrates a superior fit (R^2^ = 0.969) compared to the Freundlich isotherm (R^2^ = 0.830). It can, thus, be concluded that for the DA-800 sample, the adsorption process occurs primarily through the formation of an adsorption monolayer, however, in the case of the DA-750 sample, the formation of a multilayer takes place [17].

A more detailed examination of the data presented in Table 3 leads to the conclusion that the calculated values of q_max_ for the case of methylene blue adsorption are in substantial concordance with the values obtained experimentally. The Langmuir constant (K_L_) was found to be highest for the DA-750 sample (14.99 dm^3^/mg). This indicates that the strongest interactions occur between the dye molecule and the DA-750 biochar during the adsorption process. The R_L_ parameter for each of the tested materials oscillated between 0 and 1, indicating that the adsorption of the tested dye on the biochar samples is favourable [17]. As evidenced by the data presented in Table 3, the DA-800 sample demonstrated higher selectivity for the aqueous solution of the organic dye than the DA-750 sample. The K_F_ constant values for the obtained samples were found to be 31.21 and 17.10 mg/g(dm^3^/mg)^1/n^, respectively. A higher coefficient 1/n was obtained from the DA-800 biochar, which suggests that the system is the most heterogeneous in nature [18].

**Figure 9:**
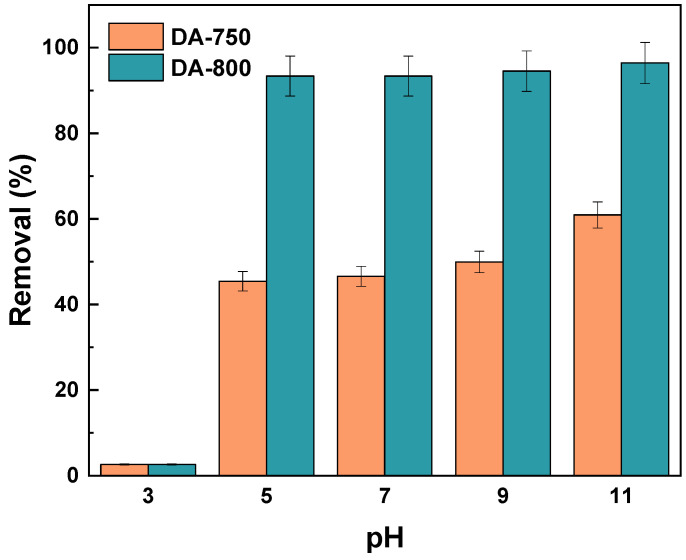
Effect of pH of the aqueous solution of dye on removal (volume of dye solution: 50 dm^3^; dye concentration: 20 mg/dm^3^, shaking speed: 250 rpm/min, time of adsorption: 24 h, temperature: 295.15 ± 1 K).

The impact of the pH of the methylene blue aqueous solution on the removal efficiency of the resulting biochar was investigated (Figure 9). The findings demonstrate that, for both samples, an increase in the pH of the dye is accompanied by an enhancement in the sorption capacity of the resulting materials. The sorption capacities of both adsorbents exhibit a pronounced decline at pH 3 in comparison to other pH values. At high pH, the sorptive material has a negative charge, which results in easier adsorption of positively charged molecules, such as methylene blue. This phenomenon has been observed in other studies [35]. Moreover, studies conducted by Mussa et al. have demonstrated that the optimal pH for methylene blue adsorption is within the range of 6–11, which corroborates the results obtained for biochar derived from fennel seeds [19].

**Figure 10:**
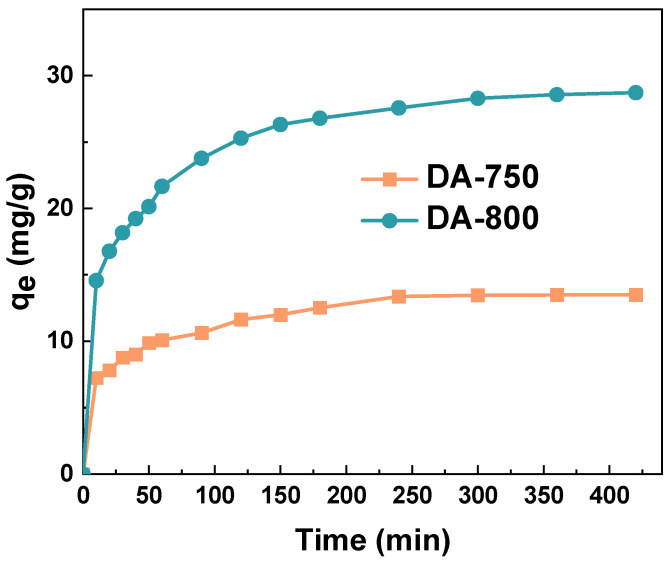
Impact of contact time on the sorption capacities of the biochar (volume of dye solution: 50 dm^3^; dye concentration: 20 mg/dm^3^, shaking speed: 250 rpm/min, temperature: 295.15 ± 1 K).

The effect of adsorbent–adsorbate contact time on the sorption capacities of the obtained biochar for methylene blue was investigated (Figure 10). Based on the presented results, it can be concluded that in the case of biochars obtained by direct activation, the equilibrium of the adsorption process is established after approximately 4 h. From an economic standpoint, this is a beneficial outcome. The findings were employed to ascertain the mechanism of dye adsorption on the obtained samples (Table 4).

**Figure 11:**
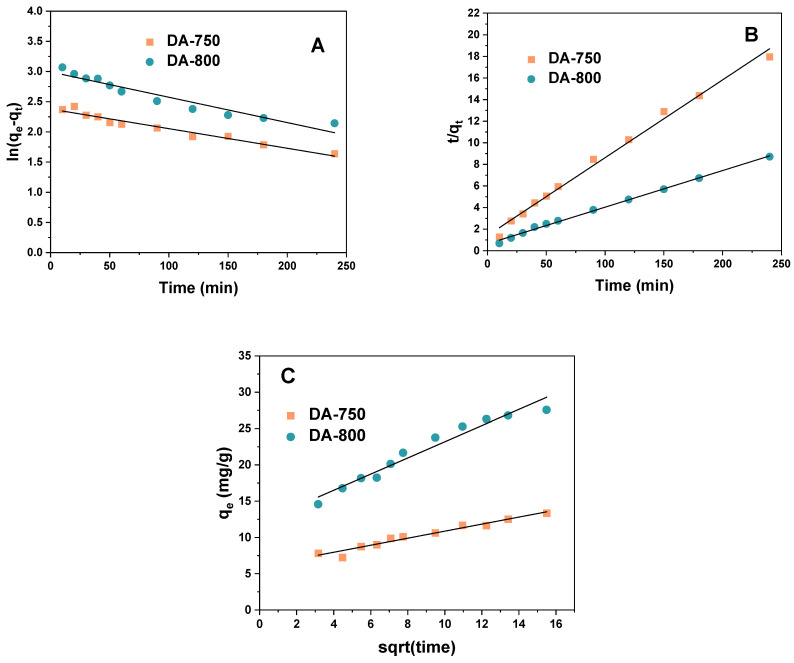
Linear fitting to (**A**) pseudo-first-order model, (**B**) pseudo-second-order model, and (**C**) intraparticle diffusion model.

Table 4 shows the parameters for three kinetic models: pseudo-first-order, pseudo-second-order, and intraparticle diffusion. The corresponding graphs for the linear kinetic models are displayed in Figure 11. A review of the data indicates that the correlation coefficients R^2^ and Adj^2^ for the pseudo-second-order model are the highest. It can, thus, be posited that the adsorption of methylene blue molecules on the sorptive materials obtained occurs according to the previously mentioned model. This is corroborated by the theoretical calculation of the sorption capacity (q_e_/cal), which is in close agreement with the experimental values. This indicates that the adsorbent is abundant in active sites [36]. The pseudo-second-order model is predicated on the assumption that the rate of adsorption of solute is directly proportional to the number of available sites on the adsorbent. Furthermore, the reaction rate is contingent upon the quantity of solute present on the surface of the adsorbent. The driving force is directly proportional to the number of active sites that are available on the adsorbent [37].

**Figure 12:**
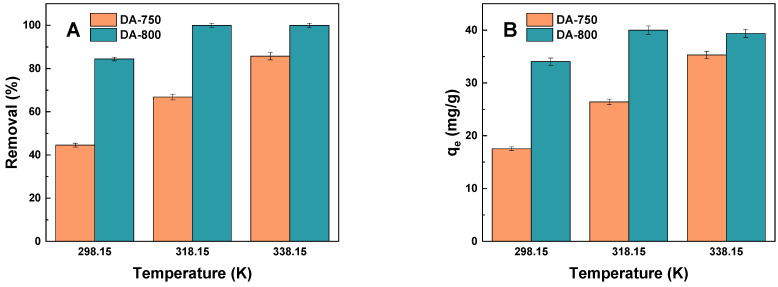
Impact of temperature of the aqueous solution of dye on (**A**) removal (%) and (**B**) sorption capacities (mg/g) (volume of dye solution: 50 dm^3^; dye concentration: 20 mg/dm^3^, shaking speed: 250 rpm/min, time of adsorption: 24 h).

Another important aspect is the thermodynamics of the process, offering insights into whether the interaction between the adsorbate and adsorbent involves physisorption or chemisorption. As the temperature rises, the system receives a greater quantity of heat, which is then transformed into kinetic energy. This results in the enhanced mobility of the dyes towards the adsorbent surface [38]. In order to ascertain the thermodynamic parameters of the adsorption process, the experiment was conducted at three different temperatures. The temperatures employed were 298.15, 318.15, and 228.15 K. As illustrated in Figure 12, the removal efficiency of the dye as well as sorption capacities demonstrated a positive correlation with increasing temperature. Nevertheless, the sorption capacity of the DA-800 biochar for the chosen dye did not demonstrate a notable enhancement (15.5% when the temperature was increased from 298.15 to 318.15 K (and further increase had no impact). From an economic standpoint, the process is advantageous due to its efficiency at room temperature and the absence of the need for additional energy input. The sorption capacity of the DA-750 adsorbent towards the methylene blue solution demonstrated a 19–22% increase when the temperature increased by 20 K.

Table 5 presents a summary of the calculated theoretical thermodynamic values for the given adsorbent samples. The values of ΔH^0^ for both samples are positive, indicating that the process of dye adsorption absorbs heat and is, therefore, endothermic in nature. The results obtained for samples DA-759 and DA-800 are 42.93 kJ/mol and 51.76 kJ/mol, respectively, which indicate that physisorption occurs [39]. A negative value of ΔG^0^ indicates a greater degree of spontaneity and a higher level of favourability for the reaction [40]. At 338 K, the lowest values of Gibbs free energy were obtained, which suggests that at that temperature the adsorption of methylene blue was the most favourable. Furthermore, the entropy change (∆S^0^) is indicative of the disorder of the system and has a substantial impact on the adsorption process, whereby dye molecules are captured and attached to the adsorbent surface [41]. The DA-800 sample exhibits a ΔS^0^ value that is over three times that of the DA-750 sample, which suggests that the adsorption process on the DA-800 sample is more disordered than that on the DA-750 sample.

### 3.3. Adsorption Mechanism

**Figure 13:**
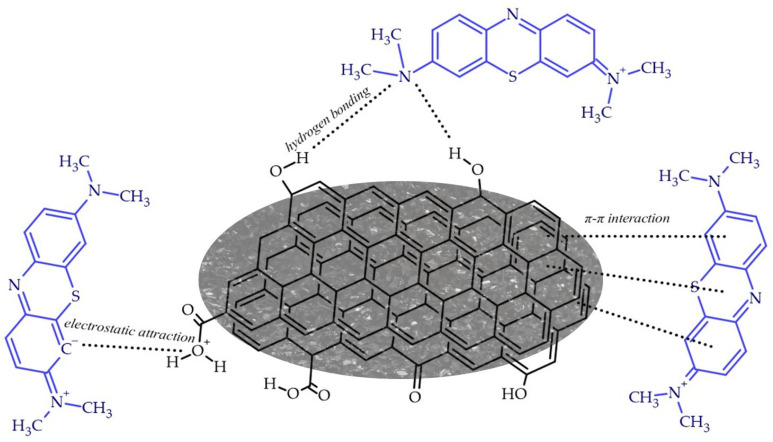
Potential interactions between methylene blue molecules and the surface of the biochar.

Figure 13 shows the potential interactions between methylene blue molecules and the surface of the biochar. The process of physisorption is dependent upon the intermolecular forces that exist between the adsorbates and the adsorbents. These forces include weak electrostatic interactions, such as dipole–dipole interactions and van der Waals forces. The process is characterised by rapid adsorption and desorption rates, and is partially reversible due to the ease of desorption [42]. π-π interactions are driven by the stacking effect between the electron-rich regions of the benzene rings on both the adsorbent and the adsorbate. The presence of three benzene rings in methylene blue renders it a feasible proposition, despite the presence of two carbons in the para positions of the middle ring. Biochar and activated carbons with high aromaticity are more likely to facilitate this type of interaction [43]. Furthermore, the presence of electronegative heteroatoms, such as nitrogen and sulphur, in the MB structure can promote hydrogen bonding, provided that the adsorbent surface contains functional groups with hydrogen atoms [44].

## 4. Conclusions

The results presented and discussed above indicate that fennel seeds may be successfully employed as a precursor for bioadsorbents obtained by direct activation for the removal of methylene blue from aqueous solutions. The physicochemical characterisation of samples DA-750 and DA-800 revealed that the temperature of activation exerts a significant influence on the specific surface area and chemical composition of the resulting adsorbents. The surface of the samples exhibited a higher content of basic groups in comparison to acidic groups. X-ray photoelectron spectroscopy analysis revealed the presence of four distinct types of carbon atoms in the DA-800 sample, whereas the DA-750 sample exhibited a single type of carbon. The analysis of the nature of methylene blue adsorption using the Langmuir and Freundlich models revealed that the data from the DA-750 sample exhibited a superior fit for the Freundlich model, whereas the data from the DA-800 sample exhibited a superior fit for the Langmuir model. This indicates the formation of a monolayer adsorbate on the DA-800 biocarbon surface during the process of methylene blue adsorption, with the subsequent formation of a multilayer on the surface of the DA-700 adsorbent. The maximum sorption capacity of the monolayer on sample DA-800 was determined to be 43 mg/g, while for sample DA-750—22 mg/g. The findings demonstrated that the sorption capacity of the examined biochar exhibited an increase as the pH value of the aqueous solution of methylene blue increased. The adsorption kinetics are most accurately described by the pseudo-second-order model. The results of the thermodynamic study indicate that the methylene blue adsorption process is physical in nature, as evidenced by the value of ΔH^0^ for sample DA-750, which was 42.93 kJ/mol, and for sample DA-800, which was 51.76 kJ/mol. Moreover, the sorption capacities of both samples increased with rising temperature, indicating that the reaction is endothermic.

## Figures and Tables

**Table 1:** Textural parameters, iodine number, and ash content of obtained biochar.

Sample	Surface Area ^1^ (m^2^/g)	Pore Volume (cm^3^/g)	Average Pore Size (nm)	Iodine Number (mg/g)	Ash Content (%)
DA-750	2.29	0.03	53.01	234	26.08
DA-800	14.60	0.09	25.75	246	28.66

^1^ Error range between 2–5%.

**Table 2:** Relative contents of elements (% At) for obtained samples based on XPS analysis.

Element	DA-750 (% At)	DA-800 (% At)
O	8.00	10.58
C	73.49	71.43
K	18.51	17.99

**Table 3:** The values of constants determined for the linear Langmuir and Freundlich models for experimental data of methylene blue.

Model	Parameters	Sample	
		DA-750	DA-800
	q_exp_ (mg/g)	22	43
Langmuir	K_L_ (dm^3^/mg)	14.99	6.38
	R_L_	0.044	0.037
	q_m_ (mg/g)	20	39
	R^2^	0.724	0.969
	Adj^2^	0.669	0.964
Freundlich	K_F_ (mg/g(dm^3^/mg)^1/n^)	17.10	31.21
	1/n	0.08	0.10
	R^2^	0.886	0.830
	Adj^2^	0.863	0.801

**Table 4:** The values of constants determined for kinetic models for experimental data.

Model	Parameters	Sample	
		DA-750	DA-800
	q_e_ (mg/g)	18	36
Pseudo-first-order	k_1_ (1/min)	1.35 × 10^−5^	1.75 × 10^−5^
	q_e/cal_ (mg/g)	11	20
	R^2^	0.956	0.923
	Adj^2^	0.952	0.915
Pseudo-second-order	k_2_ (g/mg × min)	2.16 × 10^−3^	2.24 × 10^−3^
	q_e/cal_ (mg/g)	14	30
	R^2^	0.992	0.997
	Adj^2^	0.991	0.997
Intraparticle diffusion	k_id_ (mg/g × min^1/2^)	0.48	1.11
	C (mg/g)	6.01	12.05
	R^2^	0.960	0.957
	Adj^2^	0.955	0.952

**Table 5:** Thermodynamic parameters of adsorption on the obtained biochar.

Sample	Temperature (K)	∆G^0^ (kJ/mol)	∆H^0^ (kJ/mol)	∆S^0^ (J/mol × K)
DA-750	298.15	−1.14	42.93	147.43
	318.15	−3.65		
	338.15	−7.07		
DA-800	298.15	−5.92	51.76	480.28
	318.15	−23.07		
	338.15	−24.48		

## Data Availability

Data is contained within the article.
